# A vault-prediction formula for implantable collamer lens based on preoperative parameters: a retrospective clinical study

**DOI:** 10.1186/s12886-023-03096-9

**Published:** 2023-08-08

**Authors:** Jing Yang, Hui Li, Minhui Wu, Runzhang He, Yating Nong, Zongyin Zou, Chun Zhang, Sheng Zhou

**Affiliations:** State Key Laboratory of Ophthalmology, Zhongshan Ophthalmic Center, Sun Yat-sen University, 54S Xianlie, Guangzhou, 510060 Guangdong P.R. China

**Keywords:** Implantable collamer lens, Vault prediction, Preoperative parameters

## Abstract

**Objectives:**

The aim of the present study was to predict the vault of implantable collamer lens (ICL) using a formula established based on the preoperative parameters.

**Methods:**

This retrospective study included data on 226 eyes from 114 patients(the median age and range was 27.5 ± 5.7; 18–46 years) who underwent EVO-ICL surgery between May 2021 and April 2022. Preoperative parameters, such as anterior chamber depth (ACD) horizontal angle-to-angle diameter (ATA), Crystalline lens rise (CLR), and vault (1 week after surgery) were measured by anterior segment optical coherence tomography. The axial length of eyes (AL) and white-to-white (WTW) values were measured using an IOLmaster and calipers under a slit lamp separately.

**Result:**

The mean WTW, ACD, horizontal ATA diameter, CLR, AL, and vault 1 week after surgery were 11.42 ± 0.37 mm, 3.25 ± 0.25 mm, 11.72 ± 0.44 mm, 45.78 ± 175.42 μm, 27.61 ± 1.93 mm, and 586.51 ± 254.54 μm respectively. Multivariate regression analysis showed that the ICL-width, ACD (β = 0.293), ATA (β =-0.657), CLR (β =-0.157), and the anterior chamber angle in temporal side (T:ACA) (β=-0.277) were predictors of the vault size (adjusted-R^2^ = 0.39, P < 0.001).

**Conclusion:**

The formula built based on these preoperative parameters could be used to predict the vault before surgery. The predictors indicated that the pre-operative parameters of eye such as ACD, ATA, CLR and T:ACA play an important role in choosing optimal ICL size.

## Introduction

Phakic posterior chamber lens(PC-pIOLs) are a common surgical solution for correcting myopia with few side effects. Among the different types of PC-pIOL available in the market, implantable collamer lens (ICL) that represents the most studied PC-pIOL is increasingly becoming a first-choice option for refractive surgery in patients with high myopia [1]. With developments in surgical techniques, low-to-moderate myopia, astigmatism, and hyperopia can also be corrected by ICL [2]. A recent review revealed that the vault (the distance between the anterior crystalline lens surface and ICL) was a significant parameter for determining the success or failure of ICL implants. A lower vault was shown to be associated with cataracts [3], whereas a higher vault was associated with angle closure glaucoma [4].

An appropriate size of ICL should be chosen based on several eye parameters for optimal vault outcomes. For example, the white-to-white (WTW) and anterior chamber depth (ACD) are used for calculating the optimal ICL size using the Online Calculation and Ordering System (OCOS) (https://ocos.staarag.ch/landing/). In addition, the sulcus-to-sulcus distance (STS), measured using high-frequency ultrasound biomicroscopy, may be ideal for ICL size calculation [5, 6]. However, repeatability based on STS measurements is not reliable [7], and invasive procedures are required to obtain these measurements. The horizontal anterior chamber angle distance (ATA) has been shown to be correlated with STS and may serve as an alternative choice to STS [8, 9]. Recently, the crystalline lens rise (CLR), the crystalline lens anterior surface protrusion, was shown to be negatively associated with the vault [5, 10]. So, even for the same ICL size, the unique eye parameters may have different effects on vault. A lot of recent multivariate models developed a lot of formula to predict the vault, but for the regional differences, the outcomes were not the same [6, 11–13].

In light of this background, the present study retrospectively analyzed the parameters for ICL to determine if there was an association between vault, CLR, and other relative parameters such as ATA and used regression analysis to obtain a formula that incorporated the CLR and ICL size to predict vault outcomes.

## Materials and methods

### Inclusion and exclusion criteria

This retrospective study included 226 eyes of 114 patients (the median age and range was 27.5 ± 5.7; 18–46 years) who underwent EVO-ICL surgery to treat myopia at Zhongshan Ophthalmic Center between May 2021 and April 2022 by two senior surgeons. The indications for ICL surgery were pre-operative refractive error of all patients recruited in this study and the data of pre-operative refractive error was presented in table 1 by spherical equivalent (SE). The inclusion criteria were: (i) corneal endothelial cell density > 2,000/mm^2^; (ii) ACD > 2.8 mm, and (iii) completed EVO-ICL surgery and follow-up in this hospital. The exclusion criteria were (i) the presence of other ocular conditions, such as cataracts, glaucoma, and corneal dystrophy; (ii) systemic diseases such as diabetes, autoimmune disease, and other diseases that can influence the parameters after surgery, and (iii) patients with missing data or who were not suitable for preoperative or postoperative examination. Based on these criteria, 226 eyes from 114 patients were selected.

**Table 1:** Preoperative parameters and the vault

Parameters	Value
No. of eyes/patients	226/114
Age, years (range, min-max)	27.6 ± 5.7 (18–46)
ACD, mm (range, min-max)	3.25 ± 0.25 (2.76–4.02)
AL, mm (range, min-max)	27.61 ± 1.93 (23.77–35.44)
ATA, mm (range, min-max)	11.72 ± 0.44 (10.68–12.99)
CLR, µm (range, min-max)	45.78 ± 175.42 (-428.50–525.10)
N:ACA (range, min-max)	54.15 ± 14.19 (24.50–106.30)
T:ACA (range, min-max)	56.94 ± 13.72 (25.70–120.00)
Pupil Size, mm (range, min-max)	4.49 ± 0.84 (2.19–6.35)
SE, D (range, min-max)	-20.00 ± 3.71 (-3.25 - -20.00)
Vault, µm (range, min-max)	586.51 ± 254.54 (77.80–1219.40)
WTW, mm (range, min-max)	11.42 ± 0.37 (10.70–12.60)
ICL-Axial (range, min-max)	46.35 ± 46.52 (0.00–145.00)
ICL-Cylinder, D (range, min-max)	-1.12 ± 1.32 (-5.00–0.00)
ICL-SE, D (range, min-max)	-12.75 ± 3.99 (-20.50 - -3.00)
ICL-Sphere, D (range, min-max)	-12.18 ± 3.67 (-3.00 - -18.00)
ICL-width, mm, n (%)	
12.10	72 eyes (31.9%)
12.60	121 eyes (53.5%)
13.20	31 eyes (13.7%)
13.70	2 eyes (0.9%)

ACD, anterior chamber depth; AL, axial length; ATA, angle-to-angle diameter; CLR, crystalline lens rise; WTW, horizontal white-to-white distance; ACA, anterior chamber angle; T:ACA, anterior chamber angle in temporal side; N:ACA, anterior chamber angle in nasal side; SE, spherical equivalent. ICL-SE, implantable collamer lens spherical equivalent

**Table 2:** Summary of multivariate linear regression

Predictors	Coefficients				
	B	Standard error	b	Significance	Adjusted-R^2^
Constant	-1875.572	468.637		< 0.001^b^	0.394
ACD	294.289	84.828	0.293	0.001^b^	
ATA	-381.759	54.148	-0.657	< 0.001^b^	
CLR	-0.223	0.107	-0.157	0.038^a^	
ICL-width	380.297	64.240	0.571	< 0.001^b^	
T:ACA	-5.017	1.299	-0.277	< 0.001^b^	

^a^P≤0.05, ^b^P≤0.001. ICL, implantable collamer lens; ACD, anterior chamber depth; ATA, angle-to-angle diameter; T:ACA, anterior chamber angle in temporal side; CLR, crystalline lens rise.

### ICL size selection

ICL sizes were selected using the Online Calculation and Ordering System provided by STAAR Surgical based on WTW distance and ACD. Both the toric EVO-ICL and non-toric EVO-ICL procedures were included.

### Preoperative and postoperative protocols

Pentacam (OCULUS, Wetzlar, Germany) was performed to exclude keratoconus, evaluate the curvature and other parameters of the cornea, roughly determine the axis of corneal astigmatism and the depth of the anterior chamber depth. A swept-source AS-OCT instrument (SS-OCT, VG200D, SVision Imaging, Ltd.) was used to measure the ACD, ATA, CLR, pupil size, postoperative vault, and the nasal (N:) and temporal (T:) anterior chamber angle (ACA). The AS-OCT scans were performed along the horizontal meridian using a single scan centered on the pupil by one operator. AL was measured using an IOLmaster700 (Carl-Zeiss company, Germany). Additionally, WTW was measured using a caliper under a slit lamp; the caliper’s lowest unit of measurement was 1 mm. All parameters were measured three times and the mean value was obtained. The parameters were defined as follows: WTW, the distance between the nasal and temporal limbus points between the white sclera; ACD, the distance between the anterior surface of the crystalline lens and the posterior surface of the cornea; The horizontal ATA, the distance between the angle recesses on the nasal and temporal sides; CLR, the anteroposterior distance between the anterior crystalline lens surface and the angle recess to angle recess line [11]; postoperative vault, the distance between the posterior surface of the ICL and the apex of the crystalline lens; ACA; and the angle between peripheral cornea and the root of the iris. The ACA in the temporal side was defined as the T:ACA and N:ACA in the nasal side.

Other parameters included were age and the size of the ICL selected using OCOS.

### Surgical protocol

Prior to surgery, compound tropicamide eye drops (Zhuo bian™; Shenyang Xing Qi Eye Drops Medicine Co., Ltd) were used for mydriasis. Surface anesthesia was performed using Proparacaine Hydrochloride (Alcaine™: s.a.ALCON-CONUVERUR n.v). The axis for corneal astigmatism was used as a marker prior to surgery. Sterile operations were employed during every single stage of the surgery. The main 3.2 mm incision was made at the steepest meridian of the cornea, then the ICL was injected into the anterior chamber using a manufacturer injector cartridge (STAAR Surgical Co.) after the viscoelastic material (Singclean™; Hangzhou Singclean Medicine Products Co., Ltd) had been placed into the anterior chamber and moved to the posterior chamber through the pupil. After correcting the position of the lens, the viscoelastic material was washed out using a balanced saline solution. After surgery, antibiotics (0.3% Ofloxacin, Tarivid™; Santen Pharmaceutic Co., Ltd) were applied topically 4 times a day for 4 weeks.

All surgeries were successful, and no operative complications, such as pupillary block or intraocular pressure rises were observed. No patients required any extra surgery or exchange of the ICL lens.

### Statistical analysis

Sample size was calculated by two-step rule-of thumb that *N* ≥ *L*/*f*^2^ and where N represented the number of size, *L* = 19 and *f*^2^ = 0.15 when predictors were 12 and the size effect is medium. SPSS version 25 (IBM Corp.) was used for statistical analysis. Data normality was tested using Schapiro-Wilk test before statistical analysis. All parameters except the proportion of ICL-width were expressed as mean ± standard deviation (SD). A multivariate linear regression (MLR) analysis was performed to identify the relationship between vault and other pre-operative parameters. The predictor variables included age, AL, ATA, ACD, WTW, CLR, and the lens parameters, and the dependent variable was the vault. P < 0.05 was considered to indicate a statistically significant difference.

## Results

### Preoperative parameters and the vault of patients

Table 1 shows the clinicopathological parameters of the patients in details: AL,ICL-Sphere, ICL-cylinder, ICL-Axial, ICL-SE, WTW ,ACD, ATA ,CLR, T:ACA ,N:ACA, pupil diameter, vault 1 week after surgery and the proportions of ICL sizes.

ACA, CLR, pupil size, AL, and the parameters of ICL were used as predictor variables, and the postoperative vault was the dependent variable. Table 2 provides a summary of the MLR.

The multivariate correlation analysis model could be described using the following formula: Dependent vault = A0 + A1 x X1 + A2 x X2 + A3 x X3+… + + An x Xn + ε, where A represents the MLR coefficients and X represents the predictor variables. The formula for vault prediction was: Vault = -1,825.6 + 380.3 x ICL width + 294.3 x ACD − 387.8 x ATA − 0.22 x CLR − 5.0 x TACA.

Based on the ICL size, the formula could be modified as follows: Vault_(12.60 μm)_ = 2,738 + 294.3 x ACD − 387.8 x ATA − 0.22 x CLR – 5.0 x T:ACA; Vault_(12.10 μm )_ = Vault_(12.60 μm)_ − 190.15; Vault_(13.20 μm)_ = Vault_(12.60 μm)_ + 228.18; or Vault_(13.70 μm)_ = Vault_(12.60 μm)_ + 418.33.

According to this formula, five preoperative parameters could be used to predict the postoperative vault.

## Discussion

The vault is the most significant parameter correlated with postoperative complications and the need for re-surgery [3, 4]. When the vault is < 250 μm, > 750 μm, or approaching the critical value (300–700 μm), careful and meticulous follow-up is recommended [3, 4]. For example, in a recent case report, Descemet membrane endothelial keratoplasty(DEMK) surgery has been described as a possible protective method to crystalline lens to avoid the risk for postoperative opacification with low vault (till 80 μm ) after Visian implantable collamer lens surgery [14]. The traditional methods used to determine vaulting and choosing the ICL size based on WTW and ACD resulted in ~ 20% of the patients being outside the accepted vaulting range after surgery [6, 15]. In light of this, studies have attempted to predict the vault. For example, Trancon et al. showed 34% vault variance [11], Lee et al. showed 37% vault variance [6], Zheng et al. showed 36% vault variance [13], and Igarashi et al. showed 41% vault variance [12]. In the present study, to determine the optimal lens size for each individual, several anatomical parameters, including CLR, T:ACA, N:ACA, and AL, as well as lens parameters were used to build a formula for vault prediction. According to the multiple regression analysis, age, WTW, ATA, ACA, CLR, pupil size, AL, and ICL parameters accounted for 39% of the vault variance.

The size of the lens, also known as the ICL width, was positively associated with the vault, similar to that observed by Lee et al. [6]. This may be explained by the horizontal compression, accounting for the difference between the ICL size and ATA [11]. A bigger compression force will apply to a bigger lens than to a smaller lens, and this would result in a bending force on the center of the lens, that is, a bigger lens will bend forward the iris more compared with a smaller lens. Thus, when choosing the size of the lens, a 12.1 mm lens will produce a lower vault than a bigger one, and this should be taken into account prior to surgery.

ATA was negatively correlated with the vault. This may be explained in a similar manner to the ICL width. ATA can be regarded as the horizontal distance of the lens. When ATA is smaller, the force required to bend the lens forward towards the iris is stronger, resulting in a higher vault. Although STS should be the ideal parameter since it represents the horizontal situation of the ICL lens, the repeatability of STS measurement is not reliable [7], and STS measurement is an invasive operation. A recent study found that the horizontal ATA, an alternative choice to STS, was shown to be better correlated with STS than WTW [7–9, 16, 17]. Thus, ATA was chosen as the transverse measure of the eye.

Regarding the ACD, a positive correlation with vault was identified similar to that observed by Alfonso et al. [18, 19]. A study by Seo et al. [19] showed the effects of ACD on the vault measured by Ultrasound biomicroscopy (UBM), showing that when the ICL size was selected following the Online Calculation and Ordering System, the postoperative vault was found to be larger than expected if the preoperative ACD was also larger than normal. Therefore, when the ICL size is chosen, the effects of the ACD should be fully considered as well.

Previous studies have highlighted the role of CLR in ICL surgery [5, 11, 20]. Trancon et al. [11] established an equation that took the CLR into account. Similarly, the NK-formula included CLR as its predictor variable due to the multivariate correlation analysis [20]. According to the present study, the CLR was negatively correlated with the vault and identified as a predictor of the vault. A higher CLR resulted in a lower vault, that is, the higher the protrusion of the anterior surface of the crystalline lens, the smaller the space between the ICL and the crystalline lens due to compression. Although Gonalez-Lopez et al. [21] found that CLR was negatively associated with ACD, an accurate linear relationship could not be defined. Thus, although CLR may not play an important role in influencing vault compared with ICL size and ATA, all of these should be taken into account.

A regression analysis by Wen et al. [13] found a linear relationship between the vault and ACA. In the present study, ACA was negatively correlated with vault, which meant eyes with a higher ACA value had a lower vault. This can be illustrated by the compression force of the iris; eyes with a larger ACA have flatter irises. When the ICL is implanted behind the iris, a flat iris can produce stronger compression, thus reducing the vault. The T:ACA was shown to play a more important role than the N:ACA in influencing the vault, and this may be explained by the inclination of ICL. It is hypothesized that if the ICL is located close to the temporal side, it may experience a larger bending force of the iris on this side, although this remains to be confirmed experimentally.

The other parameters were not significantly associated with the vault. However, it has been shown that age was negatively correlated with vault [11], and this may be explained by the fact that an increase in age may result in physiological changes in the anterior pole of the eye, such as the increase in crystalline lens thickness, which leads to an increase in CLR and narrowing of the anterior chamber thickness [22, 23]. The lens power was also shown to be negatively correlated with vault [11]. The differences in outcomes between the present study and previous studies may be due to the difference in age and the ICL-SE of the samples.

This retrospective analysis built a formula that included five preoperative parameters that accounted for 39% of the vault variance. But there are some imitations in this study, first, for the participants are all Chinese, further studies are required with additional and larger sample sizes from different ethnic origin to better confirm the parameters, predict vault and to aid in the choice of the optimal ICL size. Second, the current study is a retrospective study, so another perspective study based on the current work could be promoted to provide higher-level evidence.

In conclusion, this study established a new formula which could be used to predict the vault before ICL surgery. In addition to some parameters that were included in other formulas, this study showed that the T:ACA which was ignored before also played an important role in determining the vault.

## Data Availability

The datasets used and/or analyzed during the current study are available from the corresponding author on reasonable request.

## Abbreviations

ACA: anterior chamber angle
AL: axial length
ATA: angle-to-angle diameter
CLR: crystalline lens ris
ECD: endothelial cell density
ICL: implantable collamer lens
OCOS: Online Calculation and Ordering System
STS: sulcus-to-sulcus distance
WTW: horizontal white-to-white distance
T:ACA: anterior chamber angle in temporal side
N:ACA: anterior chamber angle in nasal side
